# Role of Artificial Intelligence and Personalized Medicine in Enhancing HIV Management and Treatment Outcomes

**DOI:** 10.3390/life15050745

**Published:** 2025-05-06

**Authors:** Ashok Kumar Sah, Rabab H. Elshaikh, Manar G. Shalabi, Anass M. Abbas, Pranav Kumar Prabhakar, Asaad M. A. Babker, Ranjay Kumar Choudhary, Vikash Gaur, Ajab Singh Choudhary, Shagun Agarwal

**Affiliations:** 1 Department of Medical Laboratory Sciences, College of Applied & Health Sciences, A’Sharqiyah University, Ibra 400, Oman; rabab.mahmoud@asu.edu.om; 2 Department of Clinical Laboratory Sciences, College of Applied Medical Sciences, Jouf University, Sakala 72388, Saudi Arabia; dr.mpathology@gmail.com (M.G.S.); anasseen@hotmail.com (A.M.A.); 3 Department of Biotechnology, School of Engineering and Technology, Nagaland University, Meriema, Kohima 797004, India; prabhakar.iitm@gmail.com; 4 Department of Medical Laboratory Sciences, College of Health Sciences, Gulf Medical University, Ajman 4184, United Arab Emirates; azad.88@hotmail.com; 5 Department of Medical Laboratory Technology, UIAHS, Chandigarh University, Chandigarh 160036, India; 6 School of Paramedics and Allied Health Sciences, Centurion University of Technology and Management, R. Sitapur 761211, India; 7 Meerabai Institute of Technology, Delhi Skill and Entrepreneurship University, New Delhi 110077, India; vikash.gaur@dseu.ac.in; 8 Department of Medical Laboratory Technology, School of Allied Health Sciences, Noida International University, Greater Noida 203201, India; ajab.singh@niu.edu.in; 9 School of Allied Health Sciences, Galgotias University, Greater Noida 203201, India

**Keywords:** artificial intelligence, HIV, personalized medicine, machine learning, precision medicine, antiretroviral therapy, multi-omics, digital twin, telemedicine

## Abstract

The integration of artificial intelligence and personalized medicine is transforming HIV management by enhancing diagnostics, treatment optimization, and disease monitoring. Advances in machine learning, deep neural networks, and multi-omics data analysis enable precise prognostication, tailored antiretroviral therapy, and early detection of drug resistance. AI-driven models analyze vast genomic, proteomic, and clinical datasets to refine treatment strategies, predict disease progression, and pre-empt therapy failures. Additionally, AI-powered diagnostic tools, including deep learning imaging and natural language processing, improve screening accuracy, particularly in resource-limited settings. Despite these innovations, challenges such as data privacy, algorithmic bias, and the need for clinical validation remain. Successful integration of AI into HIV care requires robust regulatory frameworks, interdisciplinary collaboration, and equitable technology access. This review explores both the potential and limitations of AI in HIV management, emphasizing the need for ethical implementation and expanded research to maximize its impact. AI-driven approaches hold great promise for a more personalized, efficient, and effective future in HIV treatment and care.

## 1. Introduction

Human immunodeficiency virus (HIV), the causative agent of acquired immunodeficiency syndrome (AIDS), remains a significant global public health challenge. Despite substantial advancements in antiretroviral therapy (ART) and the implementation of various preventive strategies, HIV/AIDS continues to be a leading cause of morbidity and mortality worldwide. The virus progressively weakens the immune system, and if left untreated, leads to the development of AIDS, a life-threatening condition marked by severe immunodeficiency [1].

Global efforts to control the HIV epidemic have evolved over the years, placing increasing emphasis on early diagnosis, prompt initiation of effective treatment, and addressing the socio-economic determinants that contribute to transmission and hinder care access. In alignment with the global commitment to end AIDS as a public health threat by 2030, as articulated in the United Nations General Assembly Political Declaration, there is an intensified focus on strengthening comprehensive HIV care pathways [1,2].

As of 2019, approximately 36.85 million individuals were living with HIV/AIDS globally, with an estimated 863,840 deaths attributed to the disease. The global age-standardized prevalence rate was 454.32 per 100,000 population. Notably, high age-standardized rates were observed in regions with low sociodemographic indices, while high SDI areas showed declining trends [3].

In 2023, for the first time, the majority of new HIV infections occurred outside sub-Saharan Africa, highlighting shifting epidemiological patterns. While sub-Saharan Africa achieved a 56% reduction in new infections since 2010, regions like Eastern Europe, Central Asia, Latin America, the Middle East, and North Africa experienced rising cases [3]. However, the HIV landscape continues to present complex challenges. These include a shifting epidemiological profile, rising comorbidities and mortality rates in specific populations, the emergence of new HIV subtypes and circulating recombinant forms (CRFs), the spread of drug-resistant mutations, and evolving transmission networks. Additionally, the increasing urgency to initiate immediate antiretroviral therapy upon HIV diagnosis has introduced both clinical and logistical challenges. These dynamic factors are reshaping the course of the epidemic and are expected to significantly influence global HIV prevention and treatment efforts in the coming years [1].

This work presents a novel perspective on the integration of artificial intelligence (AI) and personalized medicine to advance the management and treatment of HIV. Moving beyond traditional, generalized treatment guidelines, the study highlights the potential of AI-driven models to deliver tailored healthcare strategies based on comprehensive patient profiles, encompassing genetic, immunological, and clinical data. It explores the application of advanced AI techniques including machine learning, deep learning, and natural language processing across critical stages of HIV care, such as early diagnosis, drug resistance prediction, optimization of ART, and adherence monitoring. A distinctive strength of this work lies in its attention to the ethical deployment of AI, emphasizing the importance of incorporating fairness metrics and bias mitigation strategies to promote equitable healthcare delivery across diverse populations. The study further underscores the significance of multi-omics data integration and examines how AI methodologies can transform complex biological datasets into clinically actionable insights, thereby enabling more precise and individualized treatment decisions. In addition, the paper critically analyzes the challenges and opportunities associated with the real-world implementation of AI-powered clinical decision support systems (CDSS) in HIV care and offers practical recommendations to support future research and clinical translation. By addressing both the advancements and the persisting gaps in this evolving field, this work contributes to strengthening the foundation for AI-driven personalized medicine, with the ultimate aim of enhancing treatment outcomes and improving the quality of life for people living with HIV.

### 1.1. Global Burden and Treatment Challenges

HIV has continued to be a global health challenge as it affects millions of people from many populations. It was estimated that there were about 38 million people worldwide living with HIV in 2021 according to the World Health Organization (WHO) and in conjunction with the Joint United Nations Program on HIV/AIDS (UNAIDS) [4]. Among these, approximately 1.5 million people were newly infected in a year. Despite so much progress in medical research and public health interventions, it has remained the cause of significant morbidity and mortality, particularly in resource-poor parts of the world.

Diagnosis and testing: early diagnosis is crucial, yet 15% of people with HIV remain unaware of their status. Traditional tests like ELISA and Western blot require lab infrastructure, while rapid tests and self-testing improve access, but pose challenges in sensitivity and linkage to care. AI-driven diagnostics and molecular testing offer promising solutions.

Treatment and drug resistance: ART has transformed HIV into a manageable condition but requires lifelong adherence. Drug resistance due to genetic mutations complicates treatment, necessitating costly second-line therapies. Nearly 10% of new ART users already show resistance. Pharmacogenomics enables personalized treatment, optimizing drug efficacy and reducing adverse effects [5].

#### Challenges in Long-Term HIV Management

Drug resistance: 10% of new ART users in some regions have pre-treatment resistance.

Health complications: ART-related issues include metabolic disorders (diabetes, cardiovascular risks), neurocognitive impairments, and bone/renal complications.

Socio-economic barriers: stigma, discrimination, and financial constraints hinder access to testing and treatment, especially in low-income regions.

Long-term health impacts: prolonged ART use is linked to metabolic disorders, neurocognitive impairments, and organ toxicity. Bone and renal complications, especially from tenofovir-based regimens, necessitate alternative treatment strategies.

Socio-economic and structural barriers: stigma, discrimination, and financial constraints limit access to testing and treatment. Global initiatives like PEPFAR and the Global Fund have expanded ART availability, yet healthcare disparities persist. Policy and funding gaps hinder large-scale implementation of innovative treatments.

AI and individualized medication can possibly revolutionize HIV treatment. Predictive models will thereby optimize ART regimens. Pharmacogenomics or individualized treatments in response to efficacy is becoming popular as well. In fact, healthcare strengthening and integrated services coupled with health policy barriers are critical for the improved outcomes on the global scale. Further research and teamwork in such areas will help provide a brighter picture of the future of HIV, where both access and effectiveness of treatment will be improved [6].

### 1.2. Need for Innovation

HIV is still a worldwide health challenge and requires ongoing innovations to enhance patient outcomes. AI and personalized medicine offer game-changing solutions for better diagnostics, treatment optimization, and long-term care. Standard ART regimens based on population data cannot customize therapy according to individual variability. Present approaches fail to adequately overcome several issues such as drug resistance, adverse reactions, and monitoring viral loads in real-time.

#### AI in HIV Management

I. Early detection and diagnosis: AI analyses EHRs, imaging, and lab reports to detect HIV-related complications earlier and more accurately.
II. Predictive modeling: machine learning forecasts treatment responses and drug resistance, enabling proactive therapy adjustments.
III. AI-driven decision support: clinical systems integrate multi-omics data to tailor ART regimens and improve treatment outcomes.
IV. Patient adherence and engagement: AI-powered apps and chatbots enhance medication adherence through reminders, education, and real-time support [7,8].
V. Pharmacogenomics: genetic markers influence ART metabolism and efficacy, allowing personalized drug selection.
VI. Multi-omics integration: genomic and proteomic data reveal patient-specific disease signatures, guiding targeted interventions.
VII. Digital twin technology: virtual patient models simulate disease progression, optimizing treatment strategies before real-world application.

AI and personalized medicine are shifting HIV care from generalized to individualized approaches, ensuring precise, effective, and adaptive treatment strategies [8,9].

## 2. The Role of Artificial Intelligence in HIV Management

Machine Learning (ML): ML techniques are widely used to predict treatment responses, assess drug resistance, forecast disease progression, and assist in patient stratification for personalized medicine. Common algorithms include Random Forest (RF), Support Vector Machine (SVM), Logistic Regression (LR), and Gradient Boosting Machines (GBM). A list of AI tools used in HIV management is provided in Table 1.

### 2.1. Random Forest (RF)

Random Forest is an ensemble machine learning algorithm that combines multiple decision trees to improve prediction accuracy and prevent overfitting. In the context of HIV management, RF models are especially useful for analyzing complex and high-dimensional biomedical data, such as genetic mutations, clinical measurements, and demographic information, to support better decision-making.

Predicting HIV drug resistance: one of the key challenges in HIV treatment is the development of resistance to ART. Random Forest models can learn patterns from large datasets of viral genetic sequences (pol gene mutations) and their associated resistance profiles. Once trained, the model can predict whether a new patient’s viral strain is likely to be resistant to specific antiretroviral drugs, helping clinicians to personalize treatment regimens [16].

Forecasting treatment outcomes: Random Forest models can integrate clinical features (CD4 count, viral load, adherence patterns, and comorbidities) to predict the likelihood of successful viral suppression or treatment failure. This helps in early identification of patients at high risk of therapy failure, so their treatment can be adjusted proactively [17].

Patient risk stratification: RF models can classify patients into risk groups based on clinical and social factors, helping healthcare providers prioritize interventions such as intensified counseling, adherence support, or treatment switches [18].

### 2.2. Support Vector Machine (SVM)

Support Vector Machine is a supervised machine learning algorithm which is widely used for classification and regression tasks in biomedical research. SVM works by finding the optimal hyperplane that separates classes (e.g., drug-resistant vs. drug-sensitive viral strains) with the maximum margin, which makes it particularly effective for high-dimensional and complex datasets, such as genetic sequences and clinical parameters that are common in HIV research.

HIV drug resistance prediction: SVM has been effectively used to classify HIV-1 strains based on genotypic mutations to predict resistance to antiretroviral drugs. Identifying resistance helps clinicians to select the most effective treatment regimen for patients, reducing the likelihood of therapy failure and improving long-term management. SVM models were trained on HIV-1 pol gene mutation data to predict resistance to various antiretroviral drugs. The SVM approach showed high prediction accuracy and generalizability, proving it to be a valuable tool for guiding HIV treatment decisions in real-world clinical settings [19].

Predicting HIV disease progression: SVM can also be applied to classify patients into risk categories for HIV progression based on clinical features such as CD4 T-cell count, viral load, genetic markers, and adherence behavior. This assists in proactive patient management, helping healthcare providers modify treatment strategies early. This study evaluated SVM alongside other machine learning methods and found that SVM delivered competitive and robust performance in predicting the progression stage of HIV based on patient data [20].

Classifying patient risk from clinical notes: in real-world healthcare, a substantial amount of patient information including HIV-related health status, risk behaviors, comorbidities, and social determinants is captured as unstructured text within electronic health records (EHRs), such as physician notes, nursing reports, and discharge summaries. Manually extracting meaningful insights from these free-text sources is both labor-intensive and prone to human error. To streamline this process, NLP) techniques are employed to automatically analyze and convert unstructured text into structured, machine-readable data. Once the relevant features are extracted such as keywords and phrases like “poor ART adherence”, “unprotected sex”, “needle sharing”, “high viral load”, or “missed appointments” Support Vector Machines (SVM) can be used to classify patients into different risk categories, including the following. High risk: likely non-adherence, potential treatment failure, or new HIV infection. Moderate risk: possible barriers to treatment success or inconsistent health follow-up. Low risk: stable, adherent, and virologically suppressed patients [21].

### 2.3. Logistic Regression (LR)

LR is one of the most widely used supervised machine learning algorithms in medical research. It is particularly effective when the target outcome is binary or categorical, for example treatment success vs. treatment failure, drug resistance vs. drug sensitivity, HIV-positive vs. HIV-negative, and high-risk vs. low-risk patients. In HIV management, Logistic Regression helps clinicians and researchers predict health outcomes based on patient data, enabling better treatment planning, early intervention, and personalized patient management.

Predicting HIV treatment failure: Logistic Regression is used to identify clinical and demographic factors that increase the likelihood of HIV treatment failure. Variables such as CD4 cell count, viral load, age, adherence status, prior antiretroviral exposure are fed into the model, which estimates the probability of treatment failure. This supports proactive changes in patient care, including intensified adherence counseling, regimen switching, or closer monitoring [22,23].

Predicting HIV drug resistance: Logistic Regression can also model the likelihood of drug resistance based on genotypic mutations, helping select more effective antiretroviral therapies. Logistic Regression was used to predict resistance of HIV-1 strains to multiple antiretroviral drugs using genetic mutation profiles. The model produced a probability score for resistance, which helps clinicians avoid ineffective drugs and improve treatment outcomes [24].

Identifying risk factors for HIV infection: LR models are also applied in epidemiology to assess the relationship between behavioral, demographic, and medical factors and HIV seroconversion. This helps public health planners design prevention strategies for high-risk groups. Logistic Regression was used to predict HIV infection risk among MSM based on behavior and demographic data, helping optimize HIV screening and prevention efforts [25].

### 2.4. Gradient Boosting Machines (GBM)

GBM are a class of powerful ensemble machine learning algorithms that combine multiple weak learners (usually decision trees) into a strong predictive model. GBMs work by sequentially minimizing errors from previous models and adjusting future predictions, making them highly accurate for complex clinical datasets, such as those used in HIV research. In the context of HIV management, GBMs have shown strong performance in:

Predicting HIV treatment outcomes: GBMs can model non-linear relationships between clinical features (e.g., CD4 count, viral load, adherence patterns, comorbidities, age, and socioeconomic status) and outcomes like virological failure, immune recovery, and survival prediction. Because GBMs can handle complex interactions and missing data better than many other methods, they are highly useful in real-world HIV datasets, which often involve diverse patient populations and incomplete health records. GBMs were applied to predict the progression of HIV based on patient features like CD4 counts and viral loads. The GBM model was one of the top-performing classifiers compared to other machine learning models, helping predict which patients were at a higher risk of clinical deterioration [26].

Forecasting HIV drug resistance: GBMs have been applied to HIV genotypic and phenotypic datasets to predict the development of drug resistance. By learning complex patterns between viral genetic mutations and treatment outcomes, GBMs can predict the likelihood that a patient’s virus will develop resistance to a specific drug. This allows clinicians to adjust ART regimens before resistance emerges [26].

Patient risk stratification and early intervention: GBMs are also used in HIV risk modeling to flag individuals who are at high risk of HIV infection (in prevention models, such as for pre-exposure prophylaxis or PrEP), poor adherence to ART, or unsuppressed viral loads. This is especially helpful in population health programs and HIV surveillance. GBM-based ensemble models were used to predict which individuals in a large health system were at high risk of acquiring HIV, improving the ability to target HIV prevention strategies like PrEP [27].

### 2.5. Deep Learning in HIV Management

Deep learning (DL), a subset of ML, is particularly effective for analyzing complex biomedical datasets, such as viral genetic sequences, medical imaging, multi-omics data, and longitudinal electronic health records (EHRs). It has been widely applied to predict drug resistance mutations, model patient treatment trajectories, and detect patterns in unstructured data like medical images and clinical notes. Common deep learning architectures include convolutional neural networks (CNNs), recurrent neural networks (RNNs), and deep feedforward neural networks (DNNs). In this study, the authors employed a deep feedforward neural network, specifically a multi-layer perceptron, to predict HIV drug resistance profiles from viral genetic sequence data. Compared to traditional machine learning models, the deep learning approach significantly improved prediction accuracy by automatically learning complex features directly from the raw sequence inputs. Overall, deep learning enables the automated extraction of informative patterns from clinical and genomic data, enhancing predictive performance in tasks such as HIV drug resistance profiling and treatment outcome forecasting [28,29].

#### 2.5.1. Convolutional Neural Networks (CNNs)

CNNs are a deep learning architecture originally designed for image recognition, but they are now widely applied in various biomedical domains, including HIV research. CNNs are particularly good at recognizing complex, non-linear patterns in structured data such as medical images, genetic sequences, and molecular structures. In HIV management, CNNs help in:

HIV drug resistance prediction from genetic sequences: CNNs are used to automatically learn and extract patterns from HIV genome sequences, especially mutations in the pol gene (responsible for coding enzymes targeted by antiretroviral drugs). The CNN can distinguish between mutations associated with drug resistance versus wild-type (non-mutated) sequences, helping predict the likelihood that a patient’s virus will resist a particular treatment. In this study, the authors used CNNs to automatically analyze HIV-1 genotype sequences and predict resistance to antiretroviral drugs. The CNN model outperformed traditional machine learning models and enabled fast, automated resistance detection to guide treatment decisions [30].

Analyzing medical images in HIV-related comorbidities: CNNs can also be applied to radiology images (X-rays, CT, MRIs) to assist in managing HIV-associated conditions, such as HIV-associated neurocognitive disorders (HAND) via brain imaging, HIV-related pulmonary infections via chest X-rays, Kaposi’s sarcoma, or lymphoma detection. By improving the speed and accuracy of image-based diagnosis, CNNs help clinicians identify comorbidities early and adjust HIV treatment plans accordingly. CNNs have been applied in chest X-ray analysis to detect pulmonary infections, including HIV-related pneumonia. While the focus is often on general infection detection, these models are being increasingly adapted to HIV-specific settings [31,32,33].

HIV progression prediction from multi-omics data: CNNs can also be applied to multi-omics data (e.g., genomics, proteomics, transcriptomics) to predict HIV disease progression. Because CNNs excel at identifying hierarchical patterns, they are a strong choice for high-dimensional biological datasets [34,35].

#### 2.5.2. Recurrent Neural Networks (RNNs)

RNNs are a type of deep learning model designed to process sequential or time-series data. In healthcare, especially in diseases like HIV which require long-term monitoring, patient data are collected over time (e.g., CD4 count trends, viral loads, medication adherence, and visit history). RNNs are uniquely capable of understanding patterns over time, making them well-suited for both diagnosis support and treatment management in HIV care.

Predicting disease progression over time: RNNs can be trained on longitudinal patient data (time-stamped lab tests, vital signs, medication adherence) to predict time to treatment failure, probability of immune system recovery, and risk of HIV-related complications. Because RNNs “remember” past information, they can model how past clinical status and behaviors impact future outcomes, a key requirement in HIV management, which involves dynamic health changes. RNNs are highlighted as particularly useful for modeling electronic health records (EHRs) to predict clinical events, and have been applied in various chronic diseases, including HIV [36,37].

Medication adherence prediction: HIV treatment success is highly dependent on adherence to ART. RNNs can process historical adherence logs, medication refill data, appointment attendance, and even mobile health app data to predict whether a patient is at risk of missing doses, falling out of care, experiencing viral rebound, and early identification of adherence risks, which allows healthcare teams to intervene before treatment failure occurs [38].

Predicting HIV diagnosis from sequential health data: RNNs have also been used to predict the likelihood of HIV infection by analyzing time-stamped behavioral and clinical data, especially in population screening scenarios. For example, RNNs can process a patient’s longitudinal risk factors, lab tests, and symptoms to flag those likely to benefit from HIV testing, even if HIV has not been clinically suspected yet. The use of RNNs in processing EHR data for predictive modeling, which can be directly applied to HIV diagnosis and management workflows [39].

Monitoring HIV-associated comorbidities: patients with HIV are at increased risk for chronic conditions like cardiovascular disease, neurocognitive decline, and kidney injury. RNNs can process time-series data from wearable devices, lab tests, and imaging reports to predict the risk of comorbidities and guide preventive interventions. RNNs offer powerful predictive capabilities for HIV diagnosis and management by modeling patient health as a time-evolving process, supporting early detection of risks for treatment failure, poor adherence, or new infections, and enabling personalized data-driven clinical decisions. This makes RNN-based tools valuable both at the bedside (clinical support) and in public health surveillance for HIV [40,41].

#### 2.5.3. Deep Feedforward Neural Networks (DNNs)

DNNs are one of the most fundamental architectures in deep learning, designed to model complex relationships between input features and output predictions. In healthcare, especially in the context of HIV diagnosis, treatment optimization, and risk prediction, DNNs are highly valuable because they can process large, multi-dimensional clinical datasets and extract hidden patterns that may not be apparent to human clinicians or simpler statistical models.

Predicting HIV drug resistance: one of the most promising applications of DNNs in HIV research is the prediction of drug resistance. By training on large datasets of viral genetic sequences and known resistance profiles, DNNs can detect subtle genetic mutations, predict which antiretroviral drugs will fail for a specific patient, and support personalized therapy selection. In this study, deep feedforward neural networks were applied to HIV-1 genetic sequences to predict drug resistance with high accuracy. The DNN model outperformed traditional machine learning algorithms (e.g., support vector machines) in terms of predictive power [24,28].

Identifying high-risk individuals for early HIV diagnosis: DNNs can be trained on large-scale clinical and behavioral datasets including demographics, lab test results, sexual health history, and social determinants to predict the likelihood of HIV infection and which individuals would benefit from targeted HIV testing. Such models help public health teams optimize early detection and reduce undiagnosed cases. While this study used a mix of models including DNNs, the deep learning component significantly enhanced the accuracy of predicting HIV acquisition risk [27].

Predicting HIV treatment outcomes: DNNs are also used to predict clinical outcomes, such as CD4 T-cell recovery after antiretroviral therapy (ART) initiation, probability of virological suppression, and treatment failure risk. These predictions support clinicians in making timely treatment adjustments, improving patient monitoring, and individualizing care plans [42,43].

Integrating multi-modal data: DNNs can combine heterogeneous types of medical data such as genetic sequences, lab test results (e.g., viral load, CD4 count), imaging data (in cases of HIV-related complications), and behavioral health data. This multi-modal capacity makes them especially powerful for modeling the complex, multi-factorial nature of HIV progression and management [44,45,46].

### 2.6. Natural Language Processing in HIV Management

NLP is applied to extract useful information from EHRs, clinical notes, research literature, and social media data related to HIV risk behaviors. NLP techniques assist in identifying HIV-positive cases from unstructured clinical text, tracking disease outbreaks and adherence behavior, and automating systematic literature reviews. Common NLP techniques are named entity recognition (NER), text classification, sentiment analysis, and word embeddings (Word2Vec, BERT). This study used NLP to extract HIV-related terms and risk behaviors from electronic health record notes, allowing for the automated detection of patients at high risk of HIV infection. The system used both rule-based extraction and machine-learned classifiers to process the text data.

NLP enables automatic extraction of valuable HIV-related information from large volumes of text, aiding early diagnosis, surveillance, and personalized patient monitoring [47,48].

#### 2.6.1. Named Entity Recognition (NER)

NER is an NLP technique designed to automatically extract specific entities (e.g., diseases, drugs, symptoms, genes, and lab results) from unstructured text such as EHRs, clinical notes, research articles, social media, or public health reports. In the context of HIV care, NER systems help healthcare providers and researchers turn messy, free-text medical data into structured, usable information for clinical decision-making, early detection, and research.

Automated extraction of HIV-related clinical information: NER models can automatically scan clinical notes in EHRs to identify and extract HIV diagnosis mentions, CD4 count values, viral load test results, antiretroviral drug names, opportunistic infections (e.g., pneumocystis pneumonia, tuberculosis), and treatment side effects. This structured information can then be used to support early diagnosis by flagging risk factors, trigger alerts in clinical systems, and populate patient registries for HIV surveillance and treatment adherence monitoring. This paper highlights how NER systems extract relevant clinical entities from EHRs, including disease names (like HIV), lab results, and drug mentions, to support better data-driven diagnosis and management [49].

Early identification of at-risk patients: NER can mine large corpora of clinical or social data to flag high-risk cases. For example, if clinical notes mention repeated sexually transmitted infections (STIs), or certain social behaviors, NER can help surface this to clinicians even if HIV is not yet diagnosed, supporting earlier testing and preventive care like PrEP (pre-exposure prophylaxis) [50,51].

Drug–drug interaction and treatment optimization: HIV-positive patients often take multiple medications for both HIV and other comorbidities. NER can extract drug names from EHRs and cross-reference them with drug interaction databases. This helps avoid dangerous drug combinations, which is especially critical for HIV patients on antiretroviral therapy [52].

Literature mining for HIV research: NER is also used in HIV research to automatically extract gene names, HIV strains, drug efficacy results, and side effect reports from scientific literature, speeding up systematic reviews and meta-analyses [50,51].

#### 2.6.2. Text Classification (TC)

TC is an NLP technique where algorithms are trained to automatically categorize unstructured text into predefined classes or labels. In the context of HIV care and research, text classification helps extract meaningful patterns and insights from large volumes of free-text data, such as clinical notes, electronic health records (EHRs), scientific articles, and even social media posts.

Automated HIV diagnosis identification from clinical notes: TC systems are capable of scanning large volumes of electronic health record (EHR) notes and automatically categorizing clinical information. These systems can determine whether a patient has an HIV-positive diagnosis, assess the risk of HIV infection, and identify cases where follow-up treatment is required. In addition to improving the speed and accuracy of HIV patient identification, text classification also supports quality assurance by ensuring the correct coding of medical records. Furthermore, it can trigger automated alerts for healthcare providers to initiate appropriate testing or treatment interventions [49].

Risk patients for preventive interventions: beyond identifying confirmed HIV cases, text classification can also be employed to flag individuals at risk of infection by analyzing information embedded in clinical notes, such as behavioral observations, sexual health history, substance use patterns, and social determinants of health. By identifying these risk factors, healthcare systems can proactively target early interventions, including offering HIV screening, recommending pre-exposure prophylaxis (PrEP), and referring patients for counseling or partner testing [53].

Monitoring treatment progress and adherence: TC models can also be utilized to analyze follow-up notes, pharmacy records, and patient feedback to identify potential challenges in HIV treatment. These models are capable of flagging instances of ART non-adherence, detecting references to side effects or emerging drug resistance, and revealing social or mental health barriers to treatment as documented by healthcare providers. Early identification of such issues enables care teams to initiate timely interventions, thereby reducing the risk of treatment failure and improving overall patient outcomes [53].

Mining scientific literature for HIV research: TC is also widely applied to filter and categorize HIV-related literature, including research studies, drug resistance reports, vaccine trials, and epidemiological surveillance publications. This approach significantly accelerates the process of systematic reviews and evidence synthesis, enabling clinicians and researchers to efficiently stay informed about the latest developments in the field [53].

#### 2.6.3. Sentiment Analysis

SA is an NLP technique that is designed to detect and interpret emotions, opinions, and attitudes expressed within text data. Although it is commonly applied in domains such as customer feedback analysis, sentiment analysis also holds significant value in healthcare, particularly for assessing patient experiences, monitoring mental health status, evaluating treatment adherence, and gauging public attitudes toward HIV. In the context of HIV management, sentiment analysis serves several critical functions:

Understanding patient mental health and treatment adherence: for individuals living with HIV, emotional well-being plays a critical role in influencing treatment adherence, continued engagement in healthcare, disclosure of their status to partners or family, and the likelihood of engaging in risky behaviors such as substance use or unprotected sex. Sentiment analysis can be applied to various text sources including clinician-written EHR notes, patient self-reported feedback from surveys, patient portals, and telehealth chat logs, as well as social media posts to identify signs of emotional distress, mental health risks, or experiences of stigma. By detecting negative emotional cues such as frustration, hopelessness, or fear, sentiment analysis enables healthcare providers to initiate timely psychosocial support or counseling, which is essential for sustaining treatment adherence, improving health outcomes, and enhancing overall quality of life [54].

Monitoring HIV-related public health awareness and stigma: SA can also be applied to social media platforms (such as Twitter, Reddit, and Facebook) and online health forums, including patient communities, to monitor public sentiment related to HIV. This approach enables the tracking of reactions to HIV prevention campaigns, such as those promoting PrEP awareness, and facilitates the early detection of emerging patterns of stigma, misinformation, or fear. Insights gained from sentiment analysis can help public health agencies refine their communication strategies, improve health education efforts, reduce fear, and combat HIV-related discrimination. This study employed text analysis techniques, including sentiment analysis, to monitor public perceptions and attitudes toward HIV/AIDS through real-time Twitter conversations. The insights gained from this analysis helped identify areas where targeted health education and stigma reduction initiatives were most needed [54].

Detecting behavioral risk from text data: SA can also be integrated with other NLP techniques to analyze conversations and clinical notes for emotional cues associated with high-risk behaviors. These include indicators of risky sexual activity, depression-related treatment non-adherence, and substance abuse, all of which can elevate the risk of HIV transmission or compromise treatment outcomes. Early identification of negative emotional states enables healthcare providers to intervene proactively, addressing underlying behavioral health concerns before clinical deterioration occurs [21].

Improving patient–doctor communication: in telemedicine and digital health applications for HIV management, sentiment analysis can play a vital role by monitoring the emotional tone of patient messages in real time. This enables healthcare teams to receive alerts when a patient expresses signs of distress, fear, or hopelessness. Additionally, sentiment analysis can enhance human-–AI communication within digital HIV care platforms, improving the overall patient experience and facilitating more responsive care [21,55].

#### 2.6.4. Word Embeddings

WEs are a type of NLP technique that convert words into dense, continuous vector representations. These embeddings capture semantic meanings of words based on their context in large text corpora. Common embeddings include Word2Vec and BERT, both of which have revolutionized the way we process text in healthcare, particularly in HIV diagnosis, management, and research.

Understanding medical text and clinical notes: word embeddings, such as Word2Vec and BERT, are invaluable tools for processing unstructured clinical text from electronic health records (EHRs) and doctor–patient communications. These embeddings enable machines to grasp the meaning behind clinical jargon, medical terminology, and complex medical statements. In the context of HIV management, word embeddings assist in identifying key information, including HIV diagnoses, lab results, and treatment details within clinical notes. They also help detect HIV-related complications, such as opportunistic infections and comorbidities, as well as drug names, dosages, and potential drug interactions associated with HIV therapy. By understanding the context in which terms appear, word embeddings reduce ambiguity and enhance the accuracy of diagnosis extraction and treatment recommendations [56,57].

Enhancing HIV risk prediction: word embeddings also enhance risk prediction models by analyzing patient history and identifying patterns in symptoms, social factors, and behaviors. For instance, models like Word2Vec and BERT can effectively classify mentions of high-risk behaviors, such as unprotected sex or needle-sharing. These embeddings are particularly useful in detecting subtle risk factors for HIV acquisition that may be overlooked by traditional methods. By analyzing large volumes of patient records, as well as external sources like social media and health surveys, word embeddings facilitate the creation of comprehensive risk profiles. These profiles can then be used to guide early screening and preventive interventions, such as prescribing PrEP [58,59].

Improving HIV drug resistance prediction: word embeddings can enhance HIV drug resistance prediction models by identifying critical genetic mutations and medication regimens documented in clinical records. Embeddings like Word2Vec and BERT can be trained to recognize patterns in viral load data, mutations associated with drug resistance, and mentions of antiretroviral drugs in patient histories. By analyzing text data that include genetic sequences, lab results, and treatment history, word embeddings contribute to the development of more accurate drug resistance prediction models. This, in turn, aids in creating personalized treatment plans, ensuring that patients receive the most effective therapy based on their unique clinical profiles [28].

Enhancing HIV research through literature mining: word embeddings, such as BERT, offer significant advantages in automated literature mining, enabling researchers to extract valuable insights from an extensive body of scientific literature. For instance, BERT can be fine-tuned to automatically identify relevant studies on HIV drug resistance, vaccines, or epidemiology. It also helps classify papers into appropriate categories, such as clinical trials or observational studies, based on their content, thereby accelerating and enhancing the accuracy of systematic reviews. These capabilities are particularly beneficial for public health organizations, researchers, and clinicians, allowing them to stay current with the latest findings and apply evidence-based strategies in HIV management [60,61].

Detecting HIV-related stigma and public health trends: when combined with word embeddings like Word2Vec and BERT, sentiment analysis can be used to monitor public attitudes and detect HIV-related stigma across online forums, blogs, and social media platforms. This approach is especially valuable for understanding societal reactions to HIV prevention campaigns and assessing public perceptions of HIV testing and treatment options. By tracking sentiment and public opinion, public health organizations can adapt their strategies to reduce stigma, enhance educational efforts, and increase HIV testing and prevention initiatives [62].

### 2.7. AI Algorithms in Healthcare

**Figure 1:**
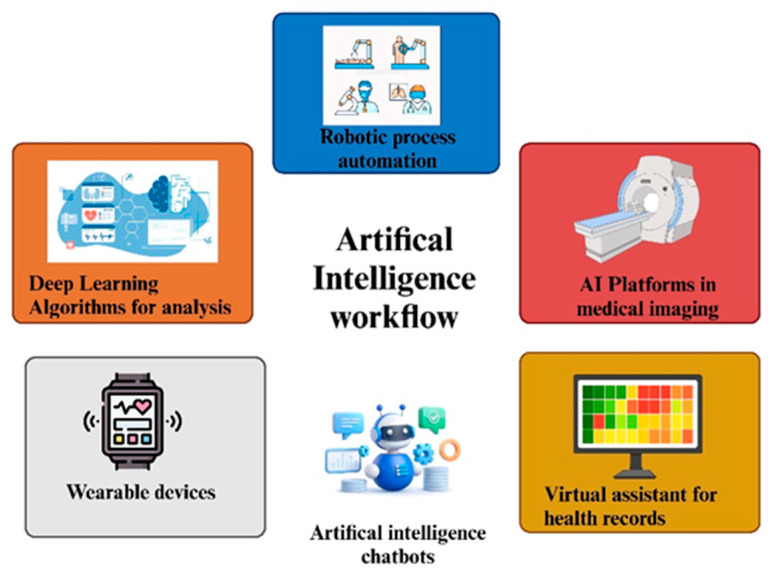
AI-driven workflow for HIV diagnosis and treatment optimization.

HIV management is being revolutionized by artificial intelligence with diagnostics, predictive analytics, and treatment optimization, and is now leaning towards artificial intelligence-enabled interventions. The AI-driven workflow is depicted in Figure 1. ML uses huge amounts of clinical and genetic data to enhance early detection, which is very important given that, for some time, HIV remains asymptomatic. As far as the traditional diagnostic methods are concerned, ELISA, as well as PCR, are both effective, but might not be available in resource-limited settings. Instead, AI-using tools like self-testing kits and mobile applications would increase accessibility by identifying key high-risk groups and recommending early testing. Similarly, AI predictive models predict disease progression through analyzing viral load, CD4 counts, and co-infections, which are used to refer patients at high risk of rapid deterioration. In addition, opportunistic infections such as tuberculosis and pneumonia can also be predicted through this model, allowing timely, preventive care. This will enable healthcare providers to give personalized, efficient, and proactive treatment, which ultimately improves patient outcomes and decreases the strain on health systems [63].

HIV management is being revolutionized by AI through drug resistance prediction, treatment optimization, and patient adherence improvement. Deep learning analyses viral genomes for identifying resistance mutations and personalized ART selection while reducing trial-and-error prescribing. AI keeps track of adherence, identifies early treatment failure, and modifies dosage in real-time to maximize the effect while limiting adverse outcomes. Smart pill dispensers and wearable devices, along with NLP-enhanced chatbots, achieve this by offering support and reminders to improve adherence. AI also contributes to public health through the analysis of epidemiological data to predict outbreaks, optimize resource allocation, and assess disease prevention strategies. It speeds-up antiviral research by identifying new compounds and mimicking immune responses for vaccines, using AI in drug discovery. NLP extracts the most from medical records in terms of consolidating clinical decision-making, especially important in telemedicine services provided at remote locations. Despite its potential benefits, however, AI adoption is not without its fair share of challenges, such as data privacy issues, algorithmic biases, and infrastructure requirements. Advancements in the futuristic phase would improve AI-fueled diagnostics, predictive modeling, and in situ monitoring of the patient, thereby enhancing HIV care, bringing it another step closer to finding a functional cure [7].

### 2.8. Predictive Modeling and Prognostication

HIV management will be transformed, with progress and treatments being predicted, establishing optimal treatment regimens, and greatly benefiting patient outcomes. AI modeling makes use of enormous amounts of data, such as clinical, demographic, and genetic information, to predict treatment responses for individuals, prospective patterns of drug resistance, and future opportunistic infections. With these machine learning algorithms, practitioners should intervene early enough to avoid or allow timely change in ART regimens and minimize the possibilities of treatment failure. Pharmacogenomic modeling will significantly affect the personalization of HIV treatment by determining the genetic factors responsible for drug metabolism and effects. ART selection has been trial and error in most cases, ultimately resulting in nonoptimal responses and adverse drug reactions. Here, AI-driven pharmacogenomics may analyze a patient’s genetic profile to fast-track the identification of effective and well-tolerated drug regimens. This approach to precision medicine drastically reduces the likelihood of toxicity and improves adherence and eventual success in long-term treatment. Epidemic forecasting methods, including data on historical and real-time epidemic trends, aid the prediction of HIV outbreaks so proactive measures such as focused testing, prevention campaigns, and allocation of health resources can be implemented. The use of AI in epidemiological surveillance allows public health authorities to limit the HIV epidemic by implementing data-driven intervention strategies. The AI-powered risk stratification models also help in identifying at-risk populations for developing context-specific prevention programs while also ensuring that high-risk populations benefit from optimal distribution of prophylactic treatment like pre-exposure prophylaxis (PrEP). In addition, ART adherence is a strong predictor of treatment outcome and AI adherence monitoring offers innovative options to track patients. Data on medication taking behaviors are monitored by wearable devices, mobile health applications, and smart pill dispensers. AI algorithms analyze the data for early signs of non-adherence and launch individualized interventions, including automated reminders, virtual counseling, and AI nudges for better behavior. This technology aims to improve patient engagement, prevent viral resistance, and encourage the maintenance of viral suppression [64].

Developed technologies such as digital twins will advance personalized management of HIV through treatment scenarios that consider the physiological and genetic peculiarities of the patient. Digital twin models combine multisource data to predict disease progression and treatment response incorporating different therapeutic conditions, thereby enabling clinicians to evolve an ART regimen ahead of a real-world decision. Such virtual experimentation optimally reduces the risk resulting from medication change and provides an overall understanding of long-term treatment effects. Even with these advances, challenges must be overcome if the fullest utilization of AI in HIV care is to be accomplished. Data privacy is one of the prominent issues, as AI models depend upon extensive patient information which must be stored securely and managed ethically. Algorithmic bias is another challenge of colossal economic importance because any AI model based on datasets that do not appropriately represent populations will make inaccurate predictions for those populations. Ease of use for clinicians and proper training for effective adoption must also be prioritized to ensure seamless integration into clinical workflows. All exciting innovations proposed for the future, including real-time analytics, NLP, and blockchain technology, could significantly enhance predictive modeling in HIV management. Real-time AI analytics would allow dynamic treatment alterations, NLP-driven chatbots would be available for instant patient support, and blockchain would facilitate safe and transparent data-sharing. With the present limitations mitigated, every innovative leap powered by AI will make further contributions toward the evolution of HIV care into something more personalized, efficient, and fair [65].

### 2.9. Diagnostic Enhancements

AI is bringing about significant changes in the HIV diagnostics industry in terms of accuracy, accessibility, and efficiency. Traditional methodologies like ELISA and PCR suffer from limitations of cost and accessibility, whereas novel AI-driven technologies like point-of-care (POC) testing, biosensing, and molecular diagnostics augment early detection possibilities. RDTs and AI-aided imaging systems can now conduct real-time and accurate identification of HIV markers; digital PCR and CRISPR-based diagnostics enhance their sensitivity. Next-generation sequencing (NGS) and multi-omics integration make it possible to select ART according to viral mutations and patient factor analyses. EHRs and NLP predict undiagnosed cases by creating maps of clinical data in search of at-risk individuals. AI chatbots and telemedicine platforms increase patient engagement, self-assessment, and remote diagnostics to facilitate care delivery to disadvantaged areas. Wearable biosensors are excellent for the continuous monitoring of HIV-positive patients. Albeit with challenges such as data privacy, algorithms’ bias, and costs, the innovations among AI will have a significant positive impact through the integration of real-time analysis, decision support tools, and predictive modeling to revolutionize HIV diagnostics, and ultimately improve health outcomes across the globe [66].

## 3. Role of Learning Paradigms

The learning paradigms such as supervised, unsupervised, and reinforcement learning used by AI models can greatly impact how these systems are applied in healthcare, including HIV management. Below is an overview of each learning type and its typical applications in AI models.

### 3.1. Supervised Learning

Supervised learning involves training a model on a labeled dataset, where the algorithm learns to associate input features with corresponding output labels. The primary objective is to develop a predictive mapping from input to output, enabling the model to make accurate predictions on new, unseen data. In the context of HIV management, supervised learning has several important applications. For disease diagnosis, models can be trained on labeled clinical datasets, such as patient records annotated with HIV status, to predict the likelihood of infection in new patients based on their clinical features. In risk prediction, supervised models can classify individuals according to behavioral and clinical risk factors, such as unprotected sex or substance use, and estimate their likelihood of acquiring HIV. Additionally, supervised learning can be applied to predict treatment outcomes, helping clinicians to assess whether a specific HIV therapy is likely to be effective for a patient, based on their medical history and treatment regimen. Commonly used supervised learning models in HIV research include Logistic Regression for binary classification of HIV status (positive or negative), Support Vector Machines (SVMs) for classifying HIV drug resistance, and deep learning architectures such as deep neural networks (DNNs) and convolutional neural networks (CNNs) for predicting HIV-related clinical outcomes [58].

### 3.2. Unsupervised Learning

Unsupervised learning refers to training models on unlabeled data, where the objective is to uncover hidden patterns, structures, or clusters without relying on predefined output labels. This approach is particularly valuable in situations where labeled outcomes are incomplete or unavailable, for example, when a dataset lacks confirmed HIV status for all patients. In the field of HIV management, unsupervised learning offers several important applications. Clustering algorithms, such as k-means or hierarchical clustering, can group patients based on shared characteristics including risk factors, age, viral load levels, or clinical profiles without requiring prior knowledge of their HIV status. Similarly, anomaly detection techniques can identify unusual patterns in clinical data, such as unexpected lab results or deviations in disease progression, which may signal early-stage HIV infection or rare complications. Commonly used unsupervised learning models in this context include k-means clustering for grouping patients with similar symptoms or risk profiles, and autoencoders, which are effective in detecting outliers or anomalies, such as rare genetic mutations or the emergence of drug-resistant HIV strains [67,68].

### 3.3. Reinforcement Learning

Reinforcement learning (RL) is a machine learning approach in which models learn optimal strategies by interacting with an environment and receiving feedback in the form of rewards or penalties. Through trial and error, the model continuously refines its actions to maximize long-term cumulative rewards. In the context of HIV management, reinforcement learning holds promising applications. One key area is the development of personalized treatment plans, where RL can help tailor ART strategies to individual patients based on their clinical characteristics and historical treatment responses. An RL model, for instance, could learn to adjust treatment regimens dynamically, identifying the sequence of interventions that is most likely to optimize health outcomes over time. Additionally, RL can be applied to the optimization of HIV prevention strategies. By modeling public health systems as dynamic environments, RL can help allocate resources such as HIV testing kits or PrEP in ways that maximize prevention impact, learning the best timing and distribution based on changing epidemiological patterns. Common examples of reinforcement learning models in this domain include deep Q-networks (DQN), which are effective for sequential decision-making in treatment pathways, and policy gradient methods, which are well-suited for learning resource allocation strategies in HIV prevention programs [69].

### 3.4. Combination of Supervised, Unsupervised, and Reinforcement Learning

In many real-world healthcare applications, combining different machine learning paradigms leads to more robust and flexible models. These hybrid approaches leverage the strengths of supervised learning (to make predictions from labeled data), unsupervised learning (to uncover hidden structures in unlabeled data), and reinforcement learning (to optimize decisions over time based on feedback). Hybrid models offer valuable opportunities for improving both diagnosis and treatment strategies. For example, an integrated diagnostic system might first apply unsupervised learning to cluster patients with similar clinical profiles, then use supervised learning to classify their individual risk of HIV based on those clusters. Once classified, reinforcement learning could guide the selection of the most effective intervention, tailored to the patient’s disease stage and treatment history. Similarly, in treatment planning, reinforcement learning can be combined with supervised models to predict which therapies are most likely to succeed for a particular patient, while unsupervised clustering helps identify subgroups of patients who share comparable treatment responses. This multi-paradigm approach enhances both the precision and adaptability of clinical decision-making in HIV care [8,70].

Algorithmic bias is a well-recognized challenge in AI, particularly in high-stakes domains such as healthcare, where biased models risk reinforcing, or even worsening, existing health disparities. In the context of HIV diagnosis and management, biases can emerge from multiple sources, including imbalanced or non-representative training datasets, human biases introduced during data labeling, and model design choices that inadvertently amplify disparities in predictions across demographic groups [71,72]. To detect such bias, both statistical and operational evaluations are essential. A common practice is to assess performance parity, ensuring that metrics such as predictive accuracy, false positive rates, and false negative rates remain consistent across patient subgroups defined by characteristics like age, gender, or ethnicity. Fairness can also be quantitatively evaluated using specialized metrics, including demographic parity, equalized odds, and disparate impact ratios, which help determine whether the model’s predictions are equitably distributed [71,72].

Mitigating algorithmic bias typically involves a combination of strategies: pre-processing techniques to balance training data, in-processing methods that apply fairness constraints during model optimization, and post-processing approaches that adjust predictions after model training. Additionally, explainability tools such as SHAP (Shapley additive explanations) and LIME (local interpretable model–agnostic explanations) play a vital role in making AI models more transparent. These tools allow researchers and clinicians to quantify how specific features, including demographic and clinical variables, influence HIV risk predictions, and to identify potential sources of bias [72].

Adversarial testing frameworks further strengthen fairness assessments by introducing synthetic or perturbed patient profiles, enabling developers to evaluate whether the model’s predictions remain stable and unbiased when sensitive attributes vary. These practices are particularly important when AI tools are deployed in clinical workflows for HIV screening and treatment planning. Addressing algorithmic bias is critical to ensuring that AI-driven HIV care supports health equity rather than perpetuating existing inequalities [73].

## 4. Personalized Medicine in HIV Treatment

### 4.1. Principles of Personalized Medicine

The use of personalized medicine in HIV management emphasizes tailoring treatment to diverse factors such as genetics, molecular biology, and lifestyle-increasing ART efficacy while alleviating side effects. Genomic profiling allows for the prediction of drug response, and thus avoids adverse reactions, such as abacavir hypersensitivity in HLA-B*5701 carriers. Individualized treatment planning considers immune response, co-infections, and demographics for the best ART choice. Multidisciplinary feedback provided by genomic, transcriptomic, proteomic, and metabolomic integration gives more information about HIV disease progression and treatment resistance. Predictive modeling, aided by artificial intelligence, analyses huge-size patient data to provide recommendations for personalized ART regimens and evaluate risks of drug resistance. Personalized medicine also reduces long-term ART-associated complications by precisely selecting drugs that suit one’s metabolic profile under the incidence of cardiovascular disease, bone loss, and organ toxicity. Digital health technologies, such as mobile applications and AI applications, improve adherence through real-time monitoring and reminders, as well as personalized support, thus boosting engagement and ultimately improving long-term outcomes. The combination of precision medicine with AI and multi-omics is differentiating HIV care into targeted, proactive, and patient-centered approaches, A list of these strategies is presented in Table 2 [74].

Telemedicine and remote monitoring can enhance personalized care for people living with HIV by providing immediate feedback on vital signs, medication adherence, and disease progression, prompting interventions at the correct time and reducing healthcare disparities. Remote diagnostics under AI detect early treatment failures, thereby allowing for the adjustment of therapy at the earliest stages. Personalized medicine plays a crucial role in AIDS eradication and vaccine development by enabling the analysis of individual immune responses, which in turn offers the possibility of targeted immunotherapies and vaccines. In this respect, AI can act as a catalyst by predicting vaccine efficacy based on genetic and molecular data. However, issues of data privacy, algorithmic bias, and healthcare accessibility should be addressed. HIPAA and GDPR compliance are invaluable as steps taken to eradicate AI biases in treatment recommendations. The incorporation of personalized medicine into clinical practices requires the formulation of provider education, standardized protocols, and scalable low-cost initiatives aimed at ensuring equal access. This becomes particularly important when considering resource-scarce settings with high rates of HIV infection [81].

### 4.2. Pharmacogenomics and Biomarker Identification

Pharmacogenomics and biomarker identification assist physicians in effecting personalized or individualized ART by allowing practitioners to convert antiretroviral therapy into personalized medicine according to genetic and molecular profiles. Thus, these advancements enable optimum drug selection, optimize treatment effectiveness, and reduce adverse drug reactions, all leading to improved patient outcomes. In analyzing genetic markers associated with drug metabolism, hypersensitivity, and toxicity, pharmacogenomics improves the aptness of antiretroviral therapy prescribed to a particular patient, thereby reducing trial-and-error prescribing and improving adherence. One clear example of pharmacogenomics in ART is that of HLA-B*5701 screening for severe hypersensitivity to abacavir. People who test positive for this genetic marker are at higher risk of life-threatening allergy, and so abacavir therapy can be avoided. Furthermore, variation in the CYP3A enzyme, which is the crucial enzyme handling protease inhibitor metabolism, affects both the efficacy and safety of these drugs. So, identifying such genetic diversity allows the clinician to readjust drug dosage or alter medications to maximize therapeutic benefit while avoiding toxicities [82].

The identification of biomarkers in HIV disease management helps keep track of the disease, predict treatment responses, and recognize drug resistance. Traditional biomarkers, such as CD4 count and viral loads, are essential in the evaluation of disease progression and immune function, while emerging biomarkers such as interleukin-6 (IL-6) and C-reactive protein (CRP) provide information on systemic inflammation, which is associated with long-term complications in the HIV population. Evaluation of these inflammatory markers will help assess the risks for comorbid conditions like cardiovascular disease, allowing early intervention. Resistance testing, both genotypic and phenotypic, takes this a step further in personalizing treatment by detecting mutations in the HIV genome that confer resistance to specific antiretroviral drugs. Genotypic testing looks for known resistance mutations by analyzing viral genetic sequences, while phenotypic tests study the laboratory effectiveness of a virus in responding to various drugs. AI-based models enhance and advance their resistance-detecting ability while collating and analyzing datasets to extrapolate on probable resistance patterns even before these will become clinically relevant. In this regard, the very possibility of anticipating these resistance patterns permits changes in therapy to be enacted early when it is felt that viral suppression is in jeopardy, thus offsetting the risk of treatment failure [82].

Biomarker discovery in HIV care has gained further advancement in its ability to integrate genomics, transcriptomics, proteomics, and metabolomics data. When all these layers of biological data are analyzed simultaneously, new therapeutic targets can be discovered with a more intricate understanding of the mechanisms of disease. This approach is greatly facilitating the development of next-generation ART regimens with greater efficacy and fewer side effects. Nonetheless, several barriers must still be negotiated in order to achieve equitable implementation of precision medicine in the area of HIV care. The cost of pharmacogenomic testing and biomarker evaluation can impede access to testing, especially in resource settings, while other challenges include ethical considerations over genetic data safety and patient confidentiality. Addressing these challenges will require developing a culture of trust that promotes confidence in the uptake of COVID-19 testing. Setting standards for genetic testing, improving access to affordable diagnostics, and developing sufficiently protective data security measures are other immediate needs that may help overcome some of these barriers [83].

### 4.3. Multi-Omics Data Integration

**Figure 2:**
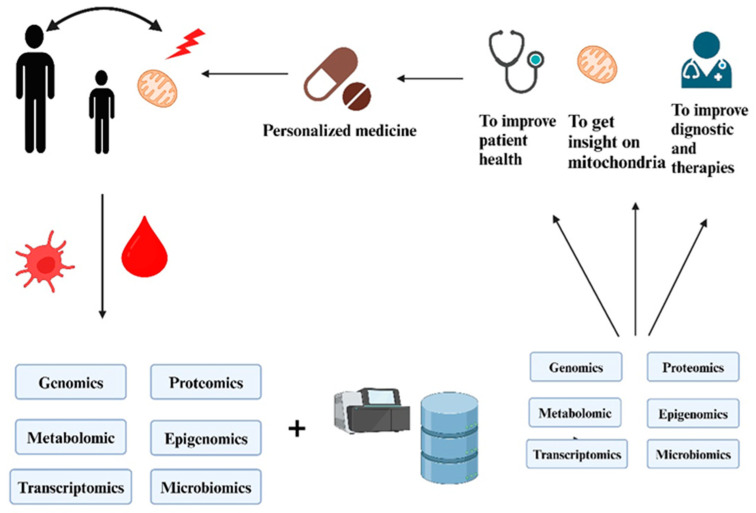
Multi-omics data integration in personalized HIV treatment.

The integration of multi-omics approaches is revolutionizing HIV treatment by combining genomics, transcriptomics, proteomics, metabolomics, epigenomics, and more to provide a comprehensive perspective on disease progression and therapeutic response. Genomics uncovers genetic factors influencing susceptibility and drug metabolism, such as the CCR5-Δ32 mutation and CYP3A variants, which are instrumental in customizing ART. Transcriptomics captures gene expression patterns associated with ART efficacy, immune response, and complications like immune reconstitution inflammatory syndrome (IRIS). Proteomics sheds light on the host proteins involved in viral replication, immune activation, and drug resistance, while also aiding in the monitoring of patients and the identification of novel therapeutic targets. Metabolomics is being leveraged to address ART-related complications, including strategies to mitigate dyslipidemia and insulin resistance. Epigenomics, in turn, investigates changes linked to viral latency and immune recovery, contributing to the development of HIV cure strategies. Artificial intelligence (AI)-driven bioinformatics further amplifies the power of multi-omics by predicting treatment outcomes, drug resistance, and adverse effects (Table 2). Despite challenges such as high costs, complexity, and data standardization, innovations in single-cell sequencing, spatial transcriptomics, and real-time monitoring hold great promise for advancing personalized HIV treatment, improving adherence, and accelerating cure and vaccine research. The mechanism underlying multi-omics data integration is illustrated in Figure 2 [84].

The integration of multi-omics data has emerged as a transformative approach for deepening our understanding of complex diseases like HIV. These rich, multi-dimensional datasets offer high-resolution insights into the molecular underpinnings of HIV infection, disease progression, immune dynamics, and treatment resistance. However, the high dimensionality, heterogeneity, and scale of such data pose significant challenges to traditional analytical methods. To navigate this complexity, artificial intelligence techniques particularly ML and DL have shown great effectiveness in identifying complex, non-linear relationships and latent biological patterns across different omics layers. Feature selection techniques help isolate biologically relevant markers by eliminating redundant or irrelevant variables, thereby enhancing model accuracy and interpretability in predicting HIV susceptibility and treatment outcomes [85,86]. Dimensionality reduction methods such as Principal Component Analysis (PCA), t-Distributed Stochastic Neighbor Embedding (t-SNE), and Uniform Manifold Approximation and Projection (UMAP) are widely used to compress complex datasets while preserving critical biological variation. These methods support improved clustering, risk stratification, and patient profiling. Additionally, integrative frameworks like Multi-Omics Factor Analysis (MOFA) and DL-based architecture enable the fusion of heterogeneous data types, resulting in more robust models and deeper biological insights. In the context of HIV diagnosis and care, these AI-powered multi-omics approaches are pivotal for early detection, predictive biomarker identification, and the discovery of new therapeutic targets, ultimately advancing precision medicine. As multi-omics data continue to grow in complexity and scale, the integration of sophisticated AI models, supported by rigorous preprocessing and dimensionality reduction, will be vital for extracting clinically actionable insights in HIV research and treatment [87,88,89].

## 5. Integration of AI and Personalized Medicine

### 5.1. Synergistic Benefits

AI and personalized medicine working hand in hand are changing the story of HIV management with great improvements in diagnosis, treatment optimization, drug-resistance prediction, and care in the long term. AI scrutinizes enormous health data, while personalized medicine customizes treatments to genetic, molecular, and lifestyle factors, ensuring ART regimens tailored to individual needs, being effective and less toxic. AI diagnostics improve early detection through the analysis of EHRs, lab results, and genetic risk factors, while self-testing applications improve accessibility. In the area of ART optimization, AI predictions of drug metabolism based on pharmacogenomic data permit the avoidance of adverse drug reactions. An example of this is the prevention of an adverse effect, namely abacavir hypersensitivity in HLA-B*5701 carriers. It detects early resistance mutations, which allow pre-emptive ART changes. Those AI-based websites really help ask the patient to comply with smart reminders and tailor-made interventions, while tracking disease progress through real-time analytical methods, and tracking along integrated multi-omics data for refining treatments. Predicting the risks and modifying therapies for managing HIV co-infections and minimizing complication risks. Regarding vaccine and cure research, AI helps hasten vaccine development by predicting potential viral latency mechanisms, while personalized medicine makes a letter a customized response. Challenges include data privacy and algorithmic bias during implementation, not to mention limited access in the resource-chain settings, making collaboration imperative for equitable enactment of AI-informed personalized HIV care [90].

### 5.2. Digital Twin Technology

HIV management is undergoing a major change due to digital twin technology, whereby virtual representations of patients can simulate how a disease progresses, patient treatment responses, and possible complications. Digital twins are capable of ART optimization and prognosis involving more predictive and personalized approaches with little trial-and-error prescribing and minimized risks for drug resistance with the integration of AI, big data, and multi-omics. Real-time patient data are continuously fed into these models, which predict immune recovery, side effects, and co-infection risks so that early interventions can be made. The ability of the digital twins to enable adherence monitoring by wearables and AI-led behavioral analysis allows personalized reminders and support to be triggered. They accelerate the drug development process in clinical trials and cure research by simulating different patient responses. Among the hindrances are data integration; computational burdens; privacy threats; and algorithmic biases, all of which require an ethical framework and cross-organizational cooperation for widespread adoption [91].

### 5.3. AI-Driven Decision Support Systems

The strongest change to HIV management yet, as conducted through an ecosystem of machine learning, natural language processing, and big data analytics, is in decision support systems, enabling the clinician to make final decisions regarding the patient; improving the outcome of ART and its management; and, in the end, better patient outcomes. These include recommendations for personalized ART regimens, predicting drug resistance and overly adjusting treatment proactively through an analysis of EHRs, genetic profiles, and viral sequences. AI-powered adherence monitoring has also been developed to locate these at-risk patients and trigger timely reminders and chatbots to improve treatment adherence. There are various AI-driven DSS for remote HIV care, co-infection management, risk stratification, and resource-limited settings, providing enhanced accessibility (Table 1). Despite improving the features, there are still hurdles, like those in data privacy, algorithmic bias in the design, and mistrust from the clinicians that require continuous refining, ethical observation, and integration strategies to realize just and effective use [15].

DSS are increasingly being developed to assist healthcare professionals by providing evidence-based recommendations, risk assessments, and real-time insights derived from complex clinical and biological datasets. In the context of HIV diagnosis and management, these systems hold considerable promises for facilitating early detection, optimizing treatment regimens, and enhancing the monitoring of patient outcomes. However, despite encouraging progress demonstrated in research environments, the translation of AI-based decision support tools into routine clinical practice for HIV care remains limited. Most existing systems are still in the developmental or conceptual stages, and have not yet reached widespread clinical implementation [92,93].

Notably, machine learning-powered predictive models have been employed to support clinicians in identifying individuals at elevated risk of poor adherence to antiretroviral therapy (ART) or those likely to experience virologic failure insights that are critical for tailoring treatment strategies and minimizing the emergence of drug resistance. These systems typically integrate diverse data sources, including electronic health records (EHRs), laboratory results, and behavioral health indicators, to compute individualized risk profiles. For instance, Marcus et al., illustrated how machine learning could be applied to large-scale, real-world clinical datasets to predict gaps in HIV care and patient non-retention, offering healthcare teams an opportunity to intervene preemptively and improve long-term treatment outcomes [15].

Despite such advancements, several barriers continue to hinder the clinical adoption of AI-driven decision support systems in HIV care. These include challenges related to model validation across diverse populations, seamless integration into existing healthcare workflows, regulatory approval, and the need for transparent and interpretable predictions. While many AI-based systems for HIV diagnosis and management have demonstrated strong performance in retrospective studies and controlled research settings, their adoption at the point of care is still rare. Moving forward, rigorous prospective validation, explainability assessments, and studies on human–AI collaboration will be essential to ensure the reliability, safety, and clinical acceptance of these tools in real-world HIV care [15,92,93].

## 6. Innovative Diagnostic and Therapeutic Approaches

### 6.1. Advanced Diagnostic Tools

AI, molecular diagnostics, biosensors, and digital health technologies have caused a revolution in HIV diagnostics, helping in early detection, treatment monitoring, and disease assessment. These technologies have increased test accuracy, reduced turnaround times, and enhanced accessibility, especially in resource-poor settings. Using AI technology and next-generation molecular testing methods, the healthcare provider can achieve earlier diagnosis of infection, enhanced tracking of disease progression, and an individual patient treatment plan. The new age of HIV involves testing through AI-based rapid tests: they couple machine learning algorithms with biosensors to eliminate human errors, while enhancing sensitivity and specificity in HIV detection. These tests study markers for large-scale inflammatory response or quantitative counting of small biological markers, like CD4, within the patient sample for precise and rapid interpretation. AI improves the interpretation of the diagnostics capture by recognizing subtle features that might otherwise go unnoticed by traditional methodologies, thus increasing the opportunities for early detection while decreasing possibilities of false-positive or false-negative results. The AI workflow can also help enable automatic analysis by decreasing dependence on skilled technicians, and thus increasing the availability of the testing facility, especially in remote and disadvantaged settings. With the advent of molecular diagnostics for next-generation applications, HIV detection and monitoring has surged to another level. With novel methodologies such as loop-mediated isothermal amplification (LAMP) and CRISPR-based approaches, tests for virus detection have become faster, cheaper, and more sensitive than conventional PCR-based methods. LAMP achieves high-speed amplification of viral residues at constant temperature without needing any special laboratory apparatus; it is fully versatile. CRISPR-based diagnostics employ gene-editing technology to detect specific HIV RNA or DNA sequences, yielding innovative point-of-care real-time access to virus detection. In this context, biosensors further the HIV diagnostic process with the real-time viral marker analysis correlation. These small and cheap devices contain biological recognition elements that identify viral proteins or nucleic acids, thus being suitable for point-of-care testing or testing from home. Technologies that will probably be available soon include wearable biosensors, measuring levels in body fluids like sweat and saliva, which continuously observe biomarkers, offering non-invasive alternatives to blood tests. Individuals will be able to check their HIV status ever more simply and frequently because of these advances, which will help manage the disease and intervene at an earlier stage [94].

Applications of state-of-the-art artificial intelligence technologies improve the diagnosis and detection of opportunistic infections and neurological complications of HIV: AI algorithms analyze medical imaging reports, which include chest X-rays and brain scans, looking for abnormalities that are found to be associated with HIV. Thus, clinicians can diagnose tuberculosis and HIV-associated neurocognitive disorders and provide treatment appropriately and in a timely manner. Liquid biopsy with the application of AI analysis provides possibilities for non-invasive monitoring of HIV treatment resistance and disease progression. Unlike traditional biopsies in tissues, liquid biopsies access circulating viral nucleic acids or immune cell markers in blood samples for real-time information on treatment effectiveness. AI-equipped computational models process complex biomarker data to find early signs of resistance to drugs and make timely changes to ART.

Modern-day imaging technologies powered by artificial intelligence are setting new standards on improving the detection of certain complications that may be related to HIV, such as opportunistic infections and neurologic disorders. AI algorithms make use of samples of medical imaging data that include chest X-rays and scans of the brain to search for abnormalities related to HIV. This informs clinicians in the diagnosis of conditions such as tuberculosis and HIV-associated neurocognitive disorders to offer timely treatment. A non-invasive method for assessing HIV treatment resistance and disease progression is liquid biopsy coupled with AI analysis. Unlike traditional biopsies in tissues, which require invasive procedures, liquid biopsies can operate by measuring circulating viral nucleic acids or immune cell markers from blood samples in real-time treatment readiness assessments. AI-based computational models observe the collection of complex biomarker data on early signs of drug resistance, allowing early changes in ART. The advent of digital health platforms and telemedicine has made HIV testing, counseling, and treatment support more accessible. This support is mobile application-assisted, meaning that users can obtain confidential medical advice and on chatbots and remote consultations to receive test results, minimizing the perceptions of stigma and enhancing patient engagement. Through such platforms, adherence may also be monitored through automated reminders and virtual counseling for better treatment adherence. There are, however, challenges with implementation and wide acceptance, including cost, accessibility, and data privacy. Admittedly, the exorbitant costs for advanced diagnostic technologies could be a limiting factor for implementation in low-income regions, which may be, in turn, caused by infrastructure constraints with regulatory hindrances. In addition, safeguarding patient data against breaches and ensuring ethical use of AI in diagnosis would require stringent policies and secure systems of data management. With investments made, new policy directions, and international collaboration in place to address these challenges to such an extent, the next step for ensuring equitable access to advanced HIV diagnostics becomes mandatory. As technology advances, better implementation of HIV detection, monitoring, and treatment interventions will be achieved with AI, biosensors, and digital health, and this will lead to the improvement of patient outcomes internationally [95].

### 6.2. Telemedicine and Wearable Technologies

Telemedicine and wearable technology have hung the door wide open for HIV management in terms of improved access into treatment, monitoring of patients, and adherence to treatment. Telemedicine will virtually eliminate barriers via real-time consultations to AI-driven chatbots and mobile health applications for the benefit of the under-served population. AI also improves cooperative decision-making through analysis of the patient database, predictive treatment requirements, and mental health services that are available through virtual counseling and therapeutic activities. Wireless devices, like smartwatches and biosensors, create a reservoir of real-time information on vital signs, medication taking, and adverse effects of ART, thus allowing for early intervention. Smart pill dispensers and biosensors help monitor levels of drugs, therefore ensuring adherence and decreasing the chances of resistance. Blockchain integration ensures data security, meaning that people are motivated to join the digital health revolution. Challenges, including limited digital access, the prominence of literacy problems in technology-related issues, inaccurate data, and compatibility with other healthcare systems, are inevitable for widespread acceptance of innovation. Some of these can be expanded, including digital infrastructure, education, and interoperability, all of which are good keys to unlock innovations in HIV care [96].

### 6.3. Predictive Analytics for Treatment Resistance

The early identification of treatment resistance, optimization of ART, and improvements in patient outcomes have been made possible by predictive analytics in HIV management. Previously, the traditional detection methods employed detected resistance only after treatment failure, but AI-assisted models predict resistance patterns using genetic, clinical, and behavioral data. Genomic analyses link mutations occurring in viruses to resistance conditions, enabling the adjustment of ART regimens long before the actual treatment failure occurs. Emerging resistance trends would be detected by longitudinal viral load measurement; modeling patient adherence will use data from pill dispensers and wearable devices to detect high-risk patients. AI-powered risk assessments consider co-infections, comorbidities associated with treatment, and potential drug interactions. Machine learning algorithms continuously update ART prescriptions according to patient history and genetic factors. Telemedicine and remote predictive analytics can further enhance early resistance detection through the addition of biosensing wearables and AI-enabled symptom tracking. Challenges faced include data quality and the integration of algorithms, privacy concerns, and algorithmic bias. The importance of ensuring data accuracy, fairness of models, and compliance with regulations is vital in maximizing all the predictive analytics that would be used by managing cases of resistance to HIV treatment [97].

## 7. Challenges and Ethical Considerations

### 7.1. Data Privacy and Security

The application of data privacy and security has become an issue of paramount importance considering how sensitive patient information is and incidents of unauthorized access, breaches, and misuse. Digital health technologies such as EHRs, telemedicine, wearable devices, and AI systems have enhanced HIV care, but pose challenges that touch upon confidentiality and data protection. Unauthorized access of HIV records might lead to stigma and discrimination, thus pressuring the implementers of mechanisms to uphold the highest security integrity, comprising multi-factor authentication, role-based access, encryption, etc. Such cyberattacks against health data highlight the need for rigorous implementation of effective cybersecurity policies like encrypted storage, use of firewalls, and periodic security audits. The law says that the data must be treated in an ethical way, with patient consent obtained, and questionnaires asking about notification of a breach sent, under regulations such as HIPAA and GDPR. To protect virtual consultations, telemedicine platforms ought to use encrypted channels for communication and to diligently identify participants. Wearable devices and mHealth apps need to embrace secure data transmission methods, while allowing user data to be anonymized or shared based upon user-controlled mechanisms. It is also possible to use AI-based analytics to enhance HIV care if strict de-identification mechanisms are implemented to protect the identity of patients from algorithmic bias configurations. Secure data-sharing frameworks, like blockchain and federated learning, enable collaborative HIV research while ensuring confidentiality. Additionally, educating patients on data security practices, consent management, and personal digital safety measures will create further strength for the HIV data privacy agenda and engender trust and integrity in healthcare systems [98].

### 7.2. Algorithmic Bias and Transparency

AI and ML are increasingly integral to HIV care. The concern about algorithmic bias and transparency matters to bridge the gap. AI decision support systems have been steering ART optimization, early detection of resistance, and improved monitoring, yet biased algorithms can lead to a bias in treatment recommendations, and thereby healthcare access. Biases are contributed via unbalanced training data, a history of disparities in medical research, and constraints certain doctors may face based on implicit biases in clinical decision-making. If AI models are being developed, not just trained on, but also evaluated using datasets that do not always reflect the representation of such underrepresented populations, then these models are vulnerable to creating very inaccurate or unfair results. Clearly, algorithmic transparency is the essence. The path followed must be structured, because many AI models work as a black box, i.e., decision-making processes that are difficult to interpret. The perspective of XAI methods, such as SHAP and LIME, enhance interpretation by revealing the factors that influenced the AI decision. Dealing with bias entails several measures such as diversified and inclusive datasets, worldwide collaborations, and routine monitoring to detect disparities so they can be addressed, and at the same time made fair. Bias audit frameworks, fairness metrics, and adversarial testing will help alleviate biases and improve fairness in the AI. There must be a preliminary accountable entity, separate from an ethical and regulatory frames, that sets certain responsibilities and demands transparency on AI training data, bias mitigation strategies, and patient rights in challenging AI-based decisions. If it tends towards fairness and transparency, AI can further healthcare improvements for people at the fastest rate [99].

### 7.3. Integration into Clinical Practice

The amalgamation of AI and digital technologies has brought about a transformation in HIV management in terms of diagnosis, optimized treatment, patient monitoring, and adherence support. AI-driven decision support systems personalize antiretroviral therapy (ART) recommendations to fine-tune treatment and eliminate trial-and-error prescriptions. AI-powered diagnostics, like rapid molecular assays and self-test apps, enable the early detection and availability of ART. Remote patient monitoring, aided by telehealth and wearable devices, permits real-time tracking of adherence to treatment and modification of therapy while ensuring data security. Nonetheless, the interoperability with electronic health records (EHRs), acceptance of the health workers, regulatory compliance, and equitable access need to be addressed. Standardizing data formats and deploying explainable AI (XAI) can help build clinicians’ confidence, while training programs for the AI will enable them to adopt it. The ethical use of AI takes into concern bias mitigation, fairness, and adherence to regulations, including HIPAA and GDPR. Bridging inequities in access within resource-poor settings will require the development of inexpensive AI tools, the enhancement of telehealth networks, and partnerships between governments, non-profits, and the tech sector. Overcoming these challenges will facilitate the use of AI as a tool for significantly improving HIV care outcomes on a global scale [100].

## 8. Future Directions and Research Opportunities

### 8.1. Clinical Trials and Prospective Studies

Evaluating new ART regimens, long-term treatment effects, and innovative strategies for treating and preventing HIV infection, such as vaccines and gene therapies, are clinical and prospective studies that have advanced contemporary research treatment for HIV. The clinical trial method follows the phasing approach. The trials start with safety studies (Phase I), followed by large-scale efficacy studies (Phase III), and end with post-marketing safety monitoring (Phase IV). The new era of trials has for long-acting ART, for instance, injectable cabotegravir and dipivefrine, which aim at improving adherence and reducing the burden of swallowing pills daily. Recent advances in prevention trials have included going beyond oral PrEP into injectables as well as vaginal ring formulations. HIV vaccine research includes changing the subject’s mode of delivery to mRNA and broadly neutralizing antibodies (bNAbs). Prospective cohort studies give real-life evidence regarding ART effectiveness and drug resistance as well as long-term health effects, such as cardiovascular risks and neurocognitive decline. Cure research uses a combination of gene editing, immune-based therapies, and latency-reversing agents to study functional HIV remission. However, there are challenges in recruiting diverse study populations or communities, the presence of ethical concerns, and post-trial treatment. Moving towards increased inclusion of possible subjects, community engagement, and greater adherence to ethical guidelines should form a basis for addressing HIV research promoting equity and making an impact [101].

### 8.2. Interdisciplinary Collaborations

Integration of the expertise of different disciplines such as medicine, AI, epidemiology, pharmacology, bioinformatics, social science, and public health is most essential in creating an opportunity to advance HIV discovery, treatment, and prevention research. New developments include long-acting ART, AI-optimized treatment with Avada Kadivar, and the creation of targeted prevention programs. Specifically, AI and machine learning applications will serve the purposes of drug discovery and patient monitoring. In the same breath, epidemiologists and social scientists should design and evaluate programs for reducing transmission and addressing healthcare barriers. Social workers and mental health professionals integrate care models that both help to adhere to ART and promote well-being. Partnerships with NGOs extend the capacity of the community in HIV education, testing, and stigma-free healthcare access. Genomics and precision medicine foster individualized regimens of ART. Cure research includes integrating virology, immunology, and gene therapy approaches to identify latency-reversing agents and CRISPR-based treatments. There are still gaps left in communication, funding, and translating research into practice. Addressing these issues in multidisciplinary interdisciplinary collaborations, including concerted research initiatives, would facilitate intense progress in HIV management and cure strategies [9].

### 8.3. Scaling and Global Health Impact

Scaling up innovations for global access presents specific challenges to maintaining and advancing HIV treatment and prevention, and coordinated efforts targeting equitable distribution of ART, PrEP, and diagnostic tools across governments, health systems, and NGOs alike are paramount. ART has, indeed, facilitated the transition of HIV into a manageable chronic condition; however, these modalities remain absent for millions due to non-affordability, disruptions in supply chains, and shortages of workers. Prevention is considered a neglected area, such as PrEP (pre-exposure prophylaxis) and harm reduction programs, often due to stigma and political impediments that require public awareness and policy reform and advocacy. Through enhancing early detection and building desired investments in healthcare and infrastructure, the adoption of rapidly innovative diagnostics such as point-of-care testing and AI-driven tools is the way to go. Significant advancements in HIV vaccine and cure research do provoke multiple logistical, regulatory, and economic challenges in potential roll-out for HIV interventions in real life. Digital health technologies, on the other hand, like telemedicine and artificial intelligence, if well-invested in connectivity and integration, may capitalize on the potential expansion of HIV care. Closing accessibility gaps through drugs, pricing, and the availability of well-trained healthcare workers poses serious challenges to scaling interventions and requires pricing agreements, an expansion of artwork, and workforce availability. To rebuild trust and enthusiasm for engagement in HIV care delivery in these settings, social and cultural impediments need to be attacked and dismantled through education and community-led programs. Global partnership and sustained investment shall be made to break through these barriers to maximize the perks of HIV innovations globally [102].

## 9. Conclusions

The convergence of artificial intelligence and personalized medicine marks a transformative shift in HIV management and treatment. Given the complexity and variability of HIV pathogenesis, traditional uniform treatment approaches often fail to address individual differences in disease progression and therapeutic response. AI technologies, especially machine learning and deep learning, enable the analysis of vast, multi-dimensional datasets generated from genomics, transcriptomics, proteomics, metabolomics, and epigenomics. This multi-omics integration uncovers predictive biomarkers, reveal hidden molecular patterns, and supports the development of personalized therapeutic strategies tailored to each patient’s unique biological makeup.

AI-enhanced personalized medicine facilitates precise risk stratification, early detection of drug resistance, and optimization of antiretroviral therapy based on genetic, immunologic, and metabolic profiles. AI-driven platforms also enable real-time monitoring of patient responses, prediction of treatment outcomes, and early identification of complications like immune reconstitution inflammatory syndrome, allowing clinicians to make timely and proactive decisions.

Despite its promise, the integration of AI and multi-omics into clinical practice faces notable challenges. These include data heterogeneity, integration complexity, ethical concerns, and the need for standardized, interoperable digital health infrastructures. Ensuring equitable access to AI-powered care—especially in resource-limited settings—is crucial to avoid widening healthcare disparities.

Looking ahead, innovations in AI algorithms, single-cell and spatial omics technologies, wearable biosensors, and real-time health monitoring are poised to further advance precision HIV care. These tools not only improve treatment adherence and patient engagement but also accelerate progress toward functional cures and effective vaccines. Ultimately, the synergy between AI and personalized medicine is redefining HIV care, shifting it toward a model that is predictive, preventive, and deeply individualized.

## Figures and Tables

**Table 1:** Role of artificial intelligence in HIV management.

S. No.	AI Application	Function	Benefits	Challenges	Ref.
1	Early detection and diagnosis	AI-powered analysis of electronic health records (EHRs) and laboratory data for early HIV detection.	Faster and more accurate diagnoses.	Data privacy concerns, need for diverse datasets.	[10]
2	Predictive modeling for treatment outcomes	Uses AI to analyze patient history and predict ART response.	Helps clinicians optimize treatment before failure occurs.	Requires high-quality training data	[11]
3	AI-driven decision support systems	Provides real-time treatment recommendations based on multi-omics data.	Enhances precision medicine and individualized ART.	Integration into clinical workflows, clinician trust	[12]
4	Adherence monitoring and engagement	AI-powered apps and wearable devices track ART adherence.	Improves patient compliance and reduces resistance.	Accessibility and user adoption.	[13]
5	Epidemiological surveillance	AI tracks HIV spread and identifies high-risk regions.	Helps policymakers allocate resources effectively.	Potential biases in predictive models.	[14]
6	Drug resistance prediction	AI analyses viral genetic sequences for early resistance mutations.	Allows proactive ART regimen adjustments.	Requires integration with genomic data.	[15]

**Table 2:** Personalized medicine strategies in HIV treatment.

S. No.	Personalized Medicine Approach	Description	Benefits	Challenges	Ref.
1	Pharmacogenomics and drug response	Genetic screening to predict ART metabolism and adverse reactions.	Minimizes toxicity and improves ART efficacy.	Cost and availability of genetic testing.	[75,76]
2	Multi-omics integration	Combines genomics, proteomics, and metabolomics to tailor treatment.	Provides a comprehensive understanding of patient variability.	Complexity of data analysis, integration challenges.	[76,77]
3	Digital twin technology	Virtual patient models simulate treatment responses.	Allows clinicians to test treatment options before real-world application.	High computational demands, data security concerns.	[77,78]
4	Biomarker identification	Uses biomarkers like inflammatory markers to assess disease progression.	Enables early intervention and targeted therapies.	Standardization of biomarker use in clinical settings.	[78,79]
5	Telemedicine and wearable tech	Remote consultations and wearable devices for continuous monitoring.	Expands access to care and enhances patient engagement.	Internet access limitations, privacy issues.	[79,80]
